# Glucagon, Metabolic Dysfunction-Associated Steatotic Liver Disease and Amino Acids in Humans and Animals without Diabetes Mellitus—An Evidence Map

**DOI:** 10.3390/life14101292

**Published:** 2024-10-12

**Authors:** Katharina Maruszczak, Pia Koren, Konrad Radzikowski, Thomas Pixner, Malte Palm Suppli, Nicolai J. Wewer Albrechtsen, Daniel Weghuber, Gabriel Torbahn

**Affiliations:** 1 Department of Pediatrics, University Hospital Salzburg, Division of Gastroenterology, Hepatology and Nutrition, Paracelsus Medical University, 5020 Salzburg, Austria; k.maruszczak@salk.at (K.M.); pia.koren@hotmail.de (P.K.); k.radzikowski@salk.at (K.R.); gabriel.torbahn@klinikum-nuernberg.de (G.T.); 2 Obesity Research Unit, University Hospital Salzburg, Paracelsus Medical University, 5020 Salzburg, Austria; tom.pixner@gmx.at; 3 Department of Pediatric and Adolescent Medicine, Salzkammergutklinikum Voecklabruck, 4864 Voecklabruck, Austria; 4 Department of Clinical Biochemistry, Copenhagen University Hospital—Bispebjerg, 2400 Copenhagen, Denmark; malte.palm.suppli@regionh.dk; 5 Center for Clinical Metabolic Research, Gentofte Hospital, University of Copenhagen, 2900 Hellerup, Denmark; nicolai.albrechtsen@regionh.dk; 6 Department of Pediatrics, Paracelsus Medical University, Klinikum Nürnberg, Universitätsklinik der Paracelsus Medizinischen Privatuniversität Nürnberg, 90471 Nuremberg, Germany

**Keywords:** MASLD, glucagon, amino acid, liver-alpha cell, pediatric, liver

## Abstract

Introduction: Health systems are confronted with not only the growing worldwide childhood obesity epidemic but also associated comorbidities. These subsequently cause variations in distinct metabolic pathways, leading to metabolic dysfunction-associated steatotic liver disease (MASLD). The aim of this evidence map is to systematically evaluate the evidence and to identify research gaps on glucagon-induced amino acid (AA) turnover and its metabolic interaction with MASLD. Methodology: A systematic literature search was conducted up to April 2023 in three electronic databases. Studies were required to include at least two of the main research areas, glucagon, AA metabolism and MASLD. Two independent reviewers screened titles and abstracts according to prespecified eligibility criteria, as well as full-text articles. Results are summarized in tables stratified by human and animal studies and study population age. Results: Thirty-four references were ultimately included. The publication years dated back to 1965 showed a great increase from 2012 to 2023. In total, there were 19 animal studies and 15 human studies. Among the human studies, except for two studies in adolescents, all the studies were conducted in adults. In human studies, the methods used to evaluate metabolic changes differed among hyperinsulinemic-euglycemic clamp and oral glucose tolerance tests. Thirteen studies focused on the metabolic effects of MASLD, while only two studies explored the interaction between MASLD, glucagon and AA metabolism in humans. The other 19 studies focused on metabolomics, beta cell function or just one topic of a research area and not on interactions between one another. Conclusion: Research on the interaction between MASLD, glucagon and AA metabolism in humans is sparse and complete lacking in pediatrics. Furthermore, longitudinal studies with a focus on hyperglucagonemia independent of diabetes but related to MASLD present an unambiguous research gap.

## 1. Introduction

Over 100 years ago, glucagon was discovered by Kimball and Murlin, who revealed a hyperglycemic factor in pancreatic extracts and called this factor “the glucose agonist” [1,2]. The central role of glucagon in glucose homeostasis has typically been examined by blocking glucagon and insulin with the hormone somatostatin, which indicates that the opposing actions of the two pancreatic hormones are important in the regulation of basal glucose production in the postabsorptive state [3]. Thus, the regulation of fasting blood glucose is balanced by the inhibitory actions of insulin and the stimulatory actions of glucagon. Initially, reported in single case reports, skin rash associated with glucagonoma was reversible under parenteral nutrition, possibly due to amino acid deficiency [4,5]. These first observations underscore the fundamental role of glucagon in amino acid (AA) metabolism, which was later verified in clamp studies [6]. Glucogenic AAs are important substrate sources for gluconeogenesis mediated by glucagon in a fasting state [7]. Furthermore, glucagon promotes catabolism of AA by stimulating hepatic ureagenesis [8,9].

In parallel to the global obesity pandemic, metabolic dysfunction-associated steatotic liver disease (MASLD), previously termed nonalcoholic fatty liver disease (NAFLD), has become the most prevalent liver disorder across the entire life span [10,11,12]. In adults with obesity-associated hepatic insulin resistance, impaired glucagon receptor signaling and disruption of the glucagon pathway are associated with hyperaminoacidemia [10,13]. Similarly, patients with liver cirrhosis were demonstrated to exhibit hyperaminoacidemia, hyperglucagonaemia and impaired ureagenesis in response to glucagon [9,14,15]. In addition, increased levels of AA were shown to stimulate glucagon secretion from pancreatic alpha cells, leading to hyperglucagonaemia and alpha cell hyperplasia [8,10]. This feedback loop was recently termed the liver-alpha cell axis (LACA) and was suggested to be a potential causal link between metabolic imbalances and glucagon secretion [8,10,13]. Accordingly, fat accumulation impairs hepatic glucagon signaling, which leads to hyperaminoacidemia [8,10,13]. Since glucagon stimulates insulin release and increases hepatic glucose production [16], hyperglucagonaemia may contribute to the enhanced β-cell responsiveness and insulin resistance known to characterize early-onset type 2 diabetes (T2D) [17]. There is substantial evidence that hyperglucagonemia induces or worsens hyperglycemia [16]. In nonhuman primates, a decrease in beta cell mass, increase in alpha cell mass, or increase in glucagon exacerbates glycemia (58,59). In humans, fasting hyperglucagonaemia and elevated glucagon secretion during the oral glucose tolerance test (OGTT) or meal consumption have been shown to contribute to hyperglycemia in patients with T2D (5,13). Furthermore, elevated fasting glucagon and impaired suppression of glucagon levels during OGTT are known to occur in individuals with obesity as well as in those with T2D (2,18–23). Thus, the role of the LACA is relevant for understanding the pathophysiology of MASLD and possibly T2D.

The aim of this evidence map is to systematically summarize the literature on the metabolic interrelationship of glucagon levels, AA metabolism and liver fat and to potentially identify research gaps on glucagon-induced AA turnover and its metabolic interaction with MASLD.

## 2. Methods

### 2.1. Reporting

We conductedconstructed an evidence map according to the Preferred Reporting Items for Systematic Reviews and Meta-Analyses (PRISMA) statement extensions for Scoping reviews (PRISMA-ScR) and for Searching (PRISMA-S) to ensure detailed reporting and implementation [18,19].

All methods were defined a priori. The protocol is available upon request.

### 2.2. Eligibility Criteria

We selected primary studies according to the following criteria:

### 2.3. Population

We considered animal and human studies. We excluded studies involving pregnant subjects or those with known liver diseases other than MASLD, diabetes mellitus, psychological morbidities or allergies.

### 2.4. Outcome

At least two of the three main research areas, MASLD and/or amino acids and/or glucagon, had to be specified as explorative parameters in the full text.

### 2.5. Study and Publication Type

We included any primary study design, e.g., randomized controlled trials (RCTs) or observational studies. Nonprimary research articles, such as conference abstracts, reviews, letters, editorials, case reports, case series and qualitative studies, were excluded. We did not set the maximum study duration, time frame or language as the selection criterion.

### 2.6. Systematic Literature Search

We searched three databases, Medline (via Ovid), Embase (via Ovid), and Web of Science (via Clarivate Analytics Web of Knowledge), from 1965 to April 2023. The search strategy is shown in Supplementary Tables S1–S3. We adapted the Medline search strategy to database-specific vocabulary for the two remaining databases (Supplementary Tables S2 and S3). No filters or restrictions except for the search in Embase were used. Here, the search results were limited to Embase (limit 37 to embase) to reduce overlap with other databases. This evidence map was generated before the new nomenclature, MASLD, was published [12]. Thus, the search referred to the old terminology NAFLD. In this manuscript, the new term MASLD will be used synonymously for NAFLD.

### 2.7. Screening and Data Extraction

All identified references were exported to the reference manager software “Endnote 21” (Clariavate Analytics). The references were deduplicated according to the proposal of Bramer et al., 2016 [20]. Two reviewers independently performed title and abstract screening. To standardize the selection of references, we piloted the process using a sample of 100 studies. Papers were further selected by screening the retrieved full texts. In addition, two reviewers independently screened the reference lists of all the included full texts for further potentially relevant articles.

### 2.8. Data Extraction and Synthesis

**Figure 1:**
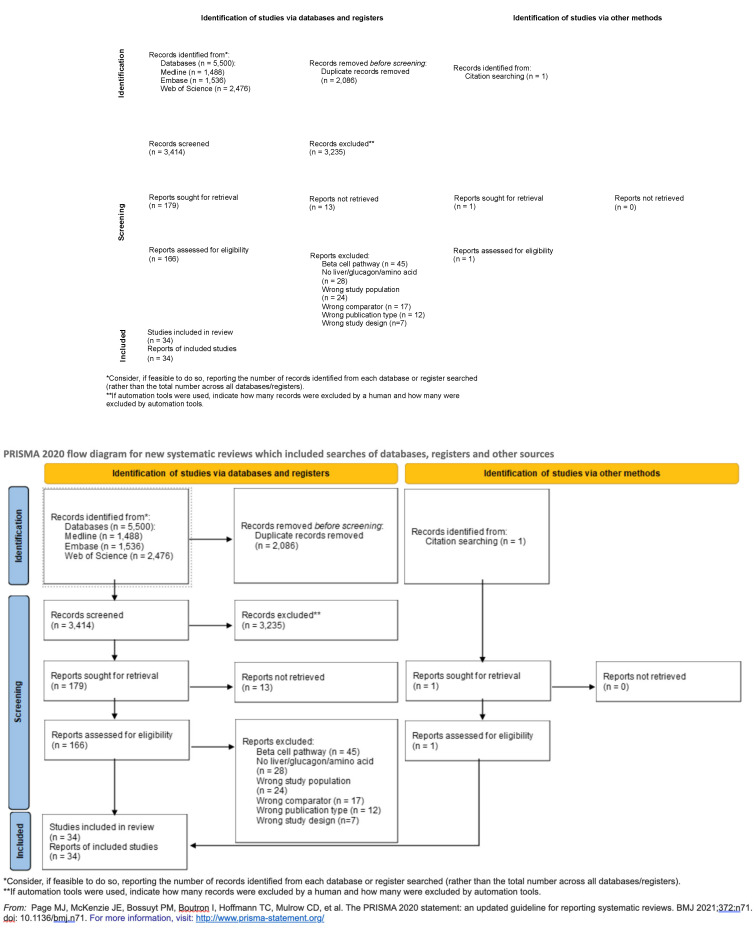
Identification of studies via databases [21,22] and study characteristics.

**Figure 2:**
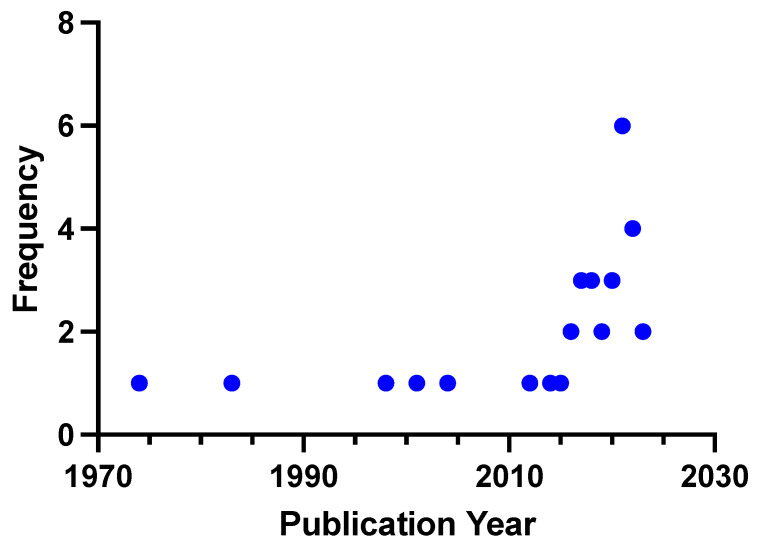
Publication frequency of the liver-alpha cell axis over the past 50 years.

**Figure 3:**
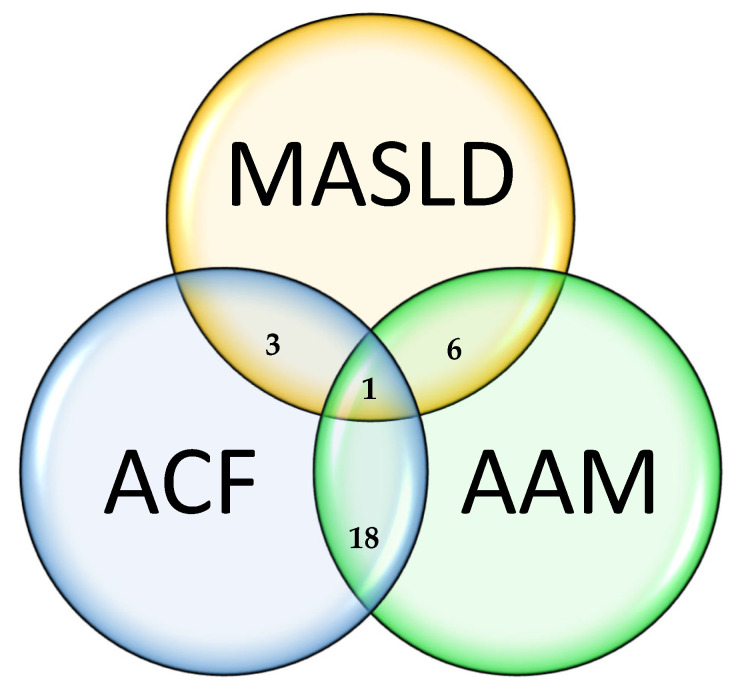
Venn diagram presenting the intersection of the three main search categories NAFLD, amino acids metabolism (AAM) and alpha cell function (ACF) for all ages including human and animal studies.

**Figure 4:**
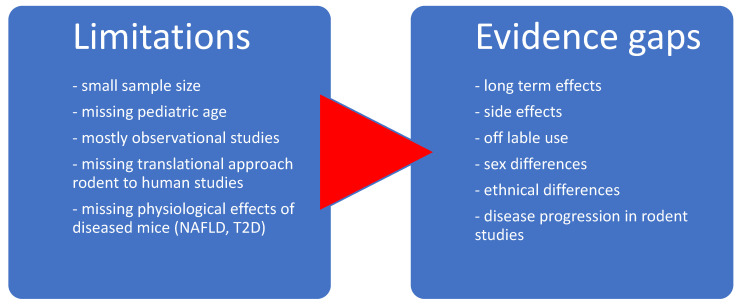
Limitations of human and animal studies illustrating evidence gaps.

We extracted the following data: study (e.g., study design, publication year, sample size, intervention, study duration and control group) or participant characteristics (e.g., comorbidities, age, sex). The descriptive data are presented in the tables. Frequencies are presented as absolute and relative numbers. We stratified the included studies into human and animal studies. To highlight the differences between the pediatric and adult cohorts in human studies, we additionally stratified patients according to age (Figure 1). To investigate the research frequency over the years, a histogram was generated (Figure 2). To visualize the relationships between the three main search areas (MASLD, AA metabolism and glucagon), a Venn diagram (Figure 3) was generated. To demonstrate the evidence gaps a graphical demonstration was designed (Figure 4).

## 3. Results

### 3.1. Systematic Literature Search

The study selection process is summarized in Figure 1. A systematic literature search of the electronic databases resulted in 5500 articles. Five duplicates were removed, and 2608 titles and abstracts were screened. One hundred thirty-seven full-text articles were retrieved, 117 of which were excluded because they did not meet our eligibility criteria. Searching the reference lists of these articles did not yield any further articles. The main reasons for the exclusion of full-text articles were a majority of the articles focused on glucose metabolism; no emphasis on one of the main search items, MASLD; AA metabolism; glucagon; wrong comparator; no full text available; and wrong study characteristics. Overall, 34 articles were included.

Table 1 and Table 2 summarize the study characteristics of the included studies. There were 18 studies conducted in animals and 15 in humans.

### 3.2. Human Studies

In the human studies (Table 1), the main study design was case control, followed by a cross-sectional design. Two RCTs and one experimental study were included [23,24,25]. The study population varied between seven and 112 study patients [25,26]. Only two studies achieved a balanced sex ratio, whereas Suppli et al., 2020 and Svane et al., 2022 included only men (n = 30) and Gar et al., 2021 included only women (n = 79) [26,27,28,29,30]. Lake et al., 2014 assessed 29 human liver samples [31]. Considering all the human studies, the ages ranged from 9–65 years. In the adult studies, the average age was 35 years. The pediatric studies included children and adolescents (9–16 years) [28,32]. Eriksen et al., (2019) and Goffredo et al., (2017) were the only studies that included overweight patients [28,33]. Battezatti et al., 1998 and Burgos et al., 2016 included only patients with a normal weight, whereas Aoki et al., 1974, Engelbrechtsen et al., 2016 and Lischka et al., 2021 included only patients with obesity [23,25,32,34,35]. Vega et al., 2021 examined overweight and obese patients [24]. Lake et al., 2014 focused on lean patients and MASLD patients, such as those with metabolic dysfunction-associated steatohepatitis (MASH) [31]. On the other hand, Grzych et al., 2020 included patients with obesity, MASH, and MASLD [26]. Svane et al., 2022 examined overweight, obese and MASLD patients [29]. Gaggini et al., 2018, Suppli et al., 2020, Eriksen et al., 2019, and Gar et al., 2021 compared lean patients with obesity with hepatic fat accumulation characteristics [27,30,33,36]. In two pediatric studies, Goffredo et al., 2021 included patients who were heterogeneous in regard to weight status and the presence of MASLD, whereas Lischka et al., 2021 included only adolescents with obesity [28,32].

### 3.3. Animal Studies

Among the 18 animal studies (Table 2), only five mentioned the total number of animals involved [37,38,39,40,41]. Most of the studies used mice in their experimental design. Gruppuso et al., 1983, Jacobs et al., 2001 and Kimball et al., 2004 chose rats for their experiments [37,42,43]. Dean et al., 2017 not only used mice but also zebrafish, and Kraft et al., 2017 had the only experimental design that included dogs [39,44]. All the animals involved were lean. Galsgaard et al., 2018 et 2020 used homozygous wild-type and glucagon receptor knockout animals [10,38]. In 2015, Solloway et al. reported not only glucagon receptor knockout but also anti-glucagon receptor knockout, as observed in lean mice [45]. Miller et al., 2018 included only GLS2 (hepatic glutaminase 2) knockout mice [46].

### 3.4. Publication Rate

After some modest initial publication activity from the 1970s to 2009, research interest in the LACA substantially increased thereafter, particularly from 2018 onward (Figure 2).

### 3.5. Alpha Cell Function

In animal studies, the role of glucagon has been described in many ways. The effects of glucagon receptor signaling on hepatic metabolism and gene expression were disrupted, especially considering AA metabolism and alpha cell proliferation [10,38,39,44,45,47]. Glucagon was also used as a control feedback mechanism for the evaluation of different metabolic mechanisms, specifically for ureagenesis and its effects on cardiometabolic risk factors such as homocysteine [38,42]. Additionally, the consequences of chronic hyperglucagonaemia and its opposing effects on insulin and glucose levels were investigated within the liver and its transcriptional consequences [37,42,43,47]. Finally, specific molecular cell activation mechanisms, for example, phosphorylation of protein kinase A, C, cAMP, and CPS1 and interactions with glucagon, were evaluated [39,43,44,45,46,47,48,49].

Aoki et al., 1974 concluded that the infusion of small amounts of glucagon resulted in a decrease in portal glucagon levels, which in turn led to a reduction in hepatic gluconeogenesis and, directly or indirectly, a compensatory increase in renal ammoniagenesis and gluconeogenesis [35]. Furthermore, a reduction in alanine and an increase in the levels of branched-chain AAs suggested that the infused glucagon also affects peripheral AA metabolism [35]. Almost 50 years later, Suppli et al., 2020 and Gar et al., 2021 suggested that hyperglucagonaemia is caused by glucagon resistance at the transcriptional and non-transcriptional levels in fat-infiltrated hepatocytes [27,30]. On a molecular level, Eriksen et al., 2019 reported that glucagon regulates the urea cycle by augmenting hepatocyte AA uptake and increasing the intramitochondrial levels of the CPS 1 activator N-acetylglutamate [33]. Furthermore, glucagon was shown to have a proteolytic effect and to promote the uptake of gluconeogenic substrates [25].

### 3.6. Amino Acid Metabolism

The main regulator of AA metabolism is insulin [10,38,39,44,45,49,50], which was first mentioned in animal studies that described a dependency between insulin and AA metabolism [39,45,49,51]. Disruption of the LACA was further shown to be characterized by the metabolic fate of specific AAs, especially gluconeogenic AAs, which are effective sources of glycogen disposition in energy metabolism [10,38,39,51]. Thus, AA catabolism is increased by glucagon, causing additional AAs to be used for gluconeogenesis [39,42,43,44,45,46,50]. Additionally, L-glutamine was identified as a potent AA that stimulates alpha-cell proliferation [44,46]. AAs promote alpha-cell proliferation ex vivo in an mTOR-dependent manner [43,44,45]. Interruption of glucagon signaling alters hepatic gene expression and increases serum AA levels [39,44,49]. Alanine-stimulated glucagon secretion enhances insulin secretion through a to b cell communication beyond what is achieved by GIPR (glucose-dependent insulinotropic polypeptide receptor) agonism in b cells alone. More specifically, Gart et al. reported that valine and isoleucine restored the damaging effects of fast food diet (FFD)-fed mice on hepatic metabolic homeostasis [40]. BCAAs counterregulate the effects of FFD through key regulators involved in energy homeostasis and lipid metabolism [40]. These beneficial metabolic effects are complemented by a decrease in immune cell-mediated liver inflammation [40]. These cellular effects were further validated by the suppression of critical inflammatory pathways known to be upregulated in MASH patients [40].

In human studies, the metabolic effects of BCAAs and the characteristics of certain AAs, such as alanine, glutamate and glutamine, were considered. BCAA levels are very sensitive to changes in insulin concentrations and are correlated with peripheral and hepatic insulin resistance [26,28,31,32,35]. Additionally, they significantly contribute to mTOR signaling and are considered risk factors for the development of T2D [23,26,31,34]. The metabolic pathway of alanine extraction by the liver is used as an index of hepatic gluconeogenesis [35]. Glutamine, on the other hand, is a major donor of NH_2_ groups and is thought to represent a marker of liver clearance that is sensitive to changes in acid–base balance [25,35]. Glutamate was shown to be involved in the LACA, as it activates AMPA/kainate receptor activity, increasing glucagon secretion [31]. As mentioned above, hyperaminoacidemia is assumed to be a consequence of hepatic glucagon resistance, potentially due to the downregulation of genes involved in hepatic AA metabolism [27,31]. Burgos et al., 2016 assessed the offspring of T2D patients to identify possible early risk factors in the alpha and beta pathways [34]. The authors showed early impairment of whole-body protein anabolism, proportionate to the degree of prevailing insulin resistance.

### 3.7. Interactions of Physiological Pathways

Most of the studies explored the interaction between AA metabolism (AAM) and alpha cell function (ACF) (n = 18). Six studies described the interaction between MASLD and AAM, whereas three studies evaluated the interaction between alpha cell function and MASLD (ACF + MASLD). Only Suppli et al., 2020 included the interaction of the three physiological pathways (MASLD + ACF + AAM) [27]. The role of LACA in nondiabetic patients and its effects on different levels of liver fat were described in detail by Suppli et al., 2020 and Gar et al., 2021 [27,30]. In vivo, Dean et al., 2017 and Solloway et al., 2015 performed basic research on the molecular interactions of the LACA in healthy rodents [44,45].

### 3.8. Liver-Alpha Cell Axis

Of the 15 human studies, two included LACA [27,30]. Both claim that alterations in this axis are already apparent at slightly increased amounts of liver fat content. The study cohort in Suppli et al., 2020 included lean controls and male nondiabetic patients with obesity and hepatic steatosis [27]. Gar et al., 2021, on the other hand, included only lean, nondiabetic and non-MASLD women [30].

In animal studies, the LACA was investigated in 10 out of 18 studies [10,39,41,43,44,45,47,48,51,52]. Additionally, the LACA was mentioned earlier in rodent studies than in human studies because of its experimental design. In addition, LACA was examined from different metabolic perspectives. The first study to evaluate the LACA was published by Kimball et al. in 2004, who hypothesized that glucagon impairs AA-induced signaling through mTOR; these authors tested this hypothesis using a perfused rat liver experimental model [43]. Kraft et al., 2017 examined whether hyperglucagonaemia redirected AA flux toward gluconeogenesis in the liver at the expense of protein synthesis [39]. The authors reported no catabolic effect of glucagon on protein synthesis in the muscle but promoted the utilization of AA rather than glucose in the liver. Data reported by Dean et al., 2017 indicated a hepatic alpha-islet cell axis where glucagon regulates serum AA concentrations and proposed that AAs, especially L-glutamine, regulate α-cell proliferation and mass via mTOR-dependent nutrient sensing [44]. Similarly, Solloway et al., (2015) suggested the occurrence of a cycle between the liver and pancreas in which glucagon-dependent clearance of AA is coupled to the regulation of alpha-cell mass [45]. Furthermore, Galsgaard et al., (2018) provided evidence that disruption of the glucagon receptor disrupts signaling and leads to increased concentrations of AA [10]. This caused the secretion of glucagon and compensatory proliferation of pancreatic alpha cells. In 2019, Galsgaard et al. investigated this relationship further and concluded that glucagon receptor signaling is more important for hepatic AA metabolism than insulin signaling is [51]. In 2022, both Maruszczak et al. and Honzawa et al. examined the LACA in association with arginine-induced glucagon secretion [41,48]. Maruszczak et al. reported that the LACA is not glucose dependent, and Honzawa et al. revealed the physiological role of the protein kinase C enzyme in glucagon secretion in pancreatic alpha cells [41,48]. Elmelund et al., 2022 and Thymiakou et al., 2022 included gene expression in the evaluation of the LACA [47,52]. Thymiakou et al., 2022 showed that the downregulation of HNFA4A gene expression has extrahepatic consequences and is connected to glucose metabolism [52]. Additionally, HNFA4A links MASLD to glucagon signaling. Elmelund et al., 2022 hypothesized that the long-term increase in glucagon receptor signaling would augment AA metabolism and ureagenesis [47]. Additionally, chronic glucagon antagonism affects the liver-alpha cell axis by causing alpha cell hyperplasia [44,45,47].

### 3.9. Omics and Gene Expression Analysis

Goffredo et al. examined the metabolomic profile of adolescents with obesity and MASLD [28]. Patients with MASLD had higher plasma levels of BCAAs, carnithin esters and long-chain phosphatidylcholine [28]. Additionally, after adjusting for confounding factors, the differences between the groups in regard to BCAAs remained significant [28]. Vega et al. observed the effects of extended glucagon exposure at various time points (up to 72 h) and performed global metabolomics and lipidomics analyses in mice [24]. Metabolomic analysis confirmed changes in glucose levels, an extensive reduction in AAs and changes in the urea cycle, whereas lipidomics revealed a reduction in triacylglycerol and diacylglycerol levels [24].

Watanabe et al. combined metabolomics and gene expression analysis to evaluate the metabolic status of the livers of GCGKO (glucagon knockout) mice [49]. The livers of GCGKO mice lacked all proglucagon-derived peptides, and altered expression of AAs, lipids, and nicotinamides was revealed [49]. Solloway et al. identified similar changes in GCGR (glucagon receptor) and anti-GCGR mice; the levels of all 20 proteinogenic AAs and urea cycle-related proteins, for example, citrulline and ornithine, were altered. Similarly, Galsgaard et al. reported that four additional AAs (alanine, glycine, threonine and serine) were strongly altered in GCGR knockout mice [10,45].

Dean et al. compared the hepatic transcriptional profiles of mice with chronic impaired glucagon signaling and acute interrupted glucagon signaling [44]. Alterations in gene expression analysis revealed alterations in both lipid and amino acid metabolism in both groups [44]. Lake et al. identified the signaling genes involved in amino acid metabolism, AA transport and the mTOR pathway [31]. The results showed that AAs, especially BCAAs and mTOR pathway genes, were associated with MASH [31]. Metabolic interactions with the progression of MASLD could be displayed not only via genetic signaling but also through changes in urea synthesis [33].

The expression of all the genes involved in the urea cycle and three additional enzymes (NAGS, GLUD1, and GLS2) directly associated with the cycle was downregulated in the MASLD groups [33]. Additionally, the expression of the amino acid transporter SLC38A3 and the glucagon receptor was significantly downregulated [33]. Elmelund et al. applied RNA sequencing to liver biopsies of mice and observed that genes related to the urea cycle, amino acid transport, amino acid metabolism and mitochondrial transporters were all affected by glucagon receptor signaling (NAG, CPS 1, SLC43A1, SLC25A15) [47]. CPS-1 results in nontranscriptional regulation of the enzyme glucagon, where chronic glucagon receptor antagonism consequently causes hyperaminoacidemia, hyperglucagonemia, alpha cell hyperplasia, decreased blood glucose levels and a decreased plasma urea index [47]. Korenfeld et al. additionally reported that 31 genes, which are directly related to AA catabolism, are transcriptionally regulated. Fasting leads to the stimulation of AA catabolism genes, leading to gluconeogenesis and ketogenesis [50].

Using RNA sequencing, Suppli et al. discovered that 352 genes were differentially regulated in MASLD males with obesity compared to lean individuals [27]. Most of the downregulated pathways were associated with the metabolism of amino acids [27]. Further examination of the specific dysregulation of amino acid metabolism revealed that several genes responsible for amino acid transporters were downregulated in MASLD patients with obesity. Additionally, the expression of *CPS1*, which encodes the important urea cycle enzyme carbamoylphosphate synthetase, was downregulated [27].

Global transcriptomic analysis of the liver was performed by Thymiakou et al., who demonstrated that the downregulation of HNF4A gene expression in the liver of mice had extrahepatic consequences and a significant impact on whole-body glucose metabolism [52]. The data revealed that HNF4A may link MASLD to glucagon [52]. HNF4A regulates ureagenesis, and altered gene expression was associated with increased serum ammonia levels in H4LivKO mice (mice lacking the HNF4A gene) [52]. The expression of genes involved in the urea cycle and the ornithine transcarbamylase gene was strongly affected in H4LivKO mice [27,52]. Silencing the HNF4A gene in mice improved glucose tolerance and insulin sensitivity, with beneficial outcomes for liver steatosis and fibrosis [52]. The HNF4A-mediated regulation of the glucagon receptor may provide a missing link between MASLD and elevated glucagon levels, as the disturbed amino acid metabolism in H4LivKO mice indicates impairment of the liver-alpha cell axis [52].

Additionally, higher levels of amino acids increase rapamycin-sensitive a-cell proliferation and the glutamine and glutamine transporter SLC38A5 [44]. These findings indicate that glutamine regulates glucagon via mTOR/FoxP-dependent control of a-cell proliferation [44]. Similarly, Solloway et al. proposed that the requirement for mTOR activation in anti-GCGR mice may link the physiological mechanism of the liver to an islet metabolic circuit controlling a-cell mass, with the molecular sensing of glucagon signaling in the liver and activation of a proliferative pathway in a-cells [45]. In this circuit, AAs act as both hepatic sensors and a-cell activators [10,33,44,45].

The molecular actions identified by Kraft et al. via RNA extraction and cDNA synthesis showed that mTOR and AMPK phosphorylation were increased in the liver at high glucagon levels, as was the hepatic expression of AA metabolism and CPS 1 [39]. However, the level of glucokinase was significantly decreased at low levels of glucagon [39]. Gart et al. showed that intervention with isoleucine or valine corrects hyperinsulinemia and reduces intrahepatic diacylglycerol levels, liver steatosis, and inflammation in Ldlr-/- mice. Leiden mice exhibit obesity-associated NASH, as demonstrated by attenuation of the lipid peroxidation marker 4-HNE [40]. Valine and isoleucine disabled crucial switches of the anabolic pathways in steatosis (SREBPF1) and consequently activated regulators of mitochondrial (PPARGC1A) and lipid metabolism (AMPK) [40]. The genetic aspects were significantly changed, and a significant reduction in histological liver inflammation and suppression of FFD-stimulated cytokine and chemokine proteins and their pathways were also observed [40].

## 4. Discussion

This evidence map summarizes clinical and nonclinical studies that investigated the metabolic interrelationship of glucagon levels, AA metabolism and liver fat content in a three-way association, the so-called liver-alpha cell axis, in patients without diabetes. In light of the substantial diversity of study methods, we could identify various evidence gaps in this research field. We present a synopsis of the mechanisms of LACA over the past 50 years and present new ideas and perspectives for future research.

The results of this evidence map identified the challenges of translational research from rodents to humans (Figure 4). Most of the animal studies identified in this review included rodents. Animal models are well suited for understanding disease mechanisms and treatment principles and for overcoming limitations of clinical trials that involve human subjects [53]. However, there are several challenges associated with clinical translation from bench to bedside that need to be considered, such as issues associated with the reproducibility of the novel discovery, metabolic rate, microbiomes and pathogens, differences due to domestication and breeding of house mice and implications for species differences [53]. Seok et al., (2013) and de Souza, (2013) compared inflammatory responses between mice and humans and found that genetically altered orthologs of mice had no relationship with their human complements [54,55,56]. Nevertheless, over the past few years, the use of mouse models, including transgenic, knockout and knockdown mouse models, has improved the understanding of the molecular determinants of MASLD [55,57]. Core metabolic enzymes that reverse the metabolic deficiencies associated with nonalcoholic fatty liver disease were identified, enabling further in-depth understanding of the pathophysiology of MASLD and allowing the development of potential new therapeutic targets [57].

Males and females are prone to respond differently to disease progression, treatment response and risk of side effects. Only two human studies in this review achieved a balanced sex ratio, and ethnicity was mentioned in only Goffredo et al., 2017 and Lischka et al., 2022 [28,32,58,59]. The sex ratio was not mentioned in any of the rodent studies, and the sample size was only stated in 3 out of 11 studies. The underreporting of the sex ratio was emphasized as one of the contributing factors to poor translation and replicability concerns, worsening preclinical research [60]. Grzych et al., 2020 was the only study acknowledging that a separate analysis of males and females is inevitable and that sex differences need to be considered when working with metabolic-based evaluations [26,59].

Furthermore, the sample size was rather small (below 16) in a quarter of the included human studies, influencing the study power. The sex-specific effect on metabolism is necessary not only in metabolic-based research but also in ethnicity. Over the years, ethnic variation in body composition has been shown to influence the development of metabolic diseases [61]. African Americans, Hispanics, American Indians, and some Pacific Islanders and Asian Americans are all at higher risk for the development of T2D than Caucasians are [61,62]. These differences may be attributed to variations in body fat patterns and lean muscle mass [63,64]. Twelve out of the 23 studies included in this review were conducted in the USA. However, only one study reported and reflected the ethnicity of the study population [11,28]. This presents an essential evidence gap and bias in the study populations, which suggests further shortcomings in the reproducibility and quality of the data.

Another study population bias is that most studies that evaluated the LACA predominantly involved diabetic patients. However, studies have shown that LACA pathophysiology is independent of T2D [13]. This finding presents a substantial evidence gap, especially when considering younger population groups, including children and adolescents, who have a growing prevalence of MASLD and hyperglucagonaemia in nondiabetic glycemic states.

### 4.1. Current LACA Knowledge in Reviews

Our study adds to the existing knowledge by summarizing the understanding of LACA via an evidence map. Currently, six reviews describe the existing evidence on LACA, each looking at it from a different perspective. The first review was published by Knop in 2018, wherein a summary of the physiological regulation of glucagon was presented, and the authors showed that the liver and gut play key roles in defining the fasting and postprandial levels of glucagon [65]. Wewer Albrechtsen et al., 2019 and Janah et al., 2019 both recognized the potential role of glucagon in lipid metabolism [66,67]. Patients who were treated with glucagon receptor antagonists had dyslipidemia and increased hepatic fat. Furthermore, a subgroup of individuals with MASLD presented a lipid-induced impairment of hepatic sensitivity, not only to insulin but also to glucagon, resulting in both hyperglucagonemia and hyperinsulinemia [66,67]. Additionally, Wewer Albrechtsen et al., (2019) reported that hyperglucagonaemia is associated with MASH independent of T2D [66]. In 2020, researchers focused more on AA catabolism [68]. The authors summarize the evidence that the interruption of glucagon signaling lowers blood glucose levels but also results in hyperglucagonemia and alpha-cell hyperplasia. Examining alpha-cell proliferation supports the pivotal role of glucagon in regulating AA metabolism. Galsgaard et al., 2020 further delineated the vicious cycle of hepatic glucagon resistance in MASLD patients and discussed the four-way association of glucagon, MASLD, lipids and AA metabolism, which leads to increased oxidative stress and worsening of MASLD [38]. The most recent review was published in 2022 by Richter MM et al. [68], who evaluated the LACA in health and disease.

### 4.2. Lack of Evidence in the Pediatric Study Population

The prevalence of childhood obesity has increased worldwide, and childhood obesity is the greatest risk factor for the development of MASLD and hyperglucagonemia [11]. MASLD in adolescents has recently been shown to substantially increase the risk of T2D in children in general, with as many as one in three children with MASLD having abnormal glucose metabolism [69,70,71]. Obesity and insulin resistance are known to contribute to this development, but beta cell and alpha cell functions, respectively, have been shown to alter insulin secretion and cause a hyperglucagonemic state in adults [72,73,74,75]. However, this has not been shown in children and adolescents. To date, only one study has compared the glucagon response to intravenous and oral glucose in young and adult patients to determine whether α-cell function differs [76]. By analyzing data from a different pediatric cohort, our group previously reported that increased hyperglucagonaemia is significantly related to increased liver fat content [77]. Lischka et al., 2021 recently reported an association between high levels of BCAAs and MASLD in children, suggesting that BCAAs could be an important link between obesity and other metabolic pathways [32]. However, the three-way association of the liver-alpha cell axis has not yet been studied in pediatric patients with MASLD.

### 4.3. Evidence Gaps

Our results, which involved a systematic review approach from three literature databases, provide a summary of the evidence throughout the identified literature. Both animal and human studies were included to represent the complete spectrum of related research. Most of the human studies had small sample sizes and particularly lacked information from the pediatric age group. The majority of the studies were cross-sectional studies with a lack of longitudinal studies, precluding interpretation of a cause–effect relationship. Limited translational approaches from animal to human studies could be identified. No research has evaluated amino acid dynamics during an OGTT or examined the effects of different types of fat distribution. Moreover, examination of glucagon dynamics in response to a glucose load or mixed meal is warranted. Furthermore, investigating the relationship between LACA and its constituents and beta cell function in regard to glucose metabolism is of interest, particularly given the longitudinal study design. In addition, none of the previous pediatric studies have combined measures of intrahepatic fat accumulation and the gold standard assessment of insulin resistance by hyperinsulinemic-euglycemic clamps. A significant downregulation of BCAA catabolism in adipose tissue, but not in liver or skeletal muscle, has been observed in patients with fatty liver disease [31,78,79]. This phenomenon has been linked to reduced mitochondrial activity or to the activation of inflammatory pathways in adipose tissue [78,79,80]. Collectively, these findings suggest the possible interaction of the LACA with inflammatory pathways. Kimball et al., 2004 and Dean et al., 2017 suggested that the disruption of the LACA is mediated by mTOR-dependent signaling [43,44]. The mTOR pathway may play a central role in AA-induced alpha-cell proliferation [43,44,45], the effect of which is most pronounced for BCAAs. Mounting evidence highlights that BCAAs in particular are associated with the risk of cardiometabolic diseases [23,26,31,34]. The evidence is most pronounced for MASLD/MASH syndrome development [28,31,32,40] and insulin resistance [26,28,31,32,35]. Interestingly, Pedersen et al. reported that after weight loss due to bariatric surgery, hyperglucagonaemia resolved, as did MASLD, but that this effect persisted in individuals with MASH despite improved insulin resistance and inflammation. They concluded that dysregulation in the LACA may persist in MASH [81]. Moreover, GLP-1 receptor signaling seems to promote insulin sensitivity and lipid accumulation via mTOR [82,83,84]. However, evidence on the extent to which recently emerged GLP-1 receptor agonists may influence this pathway is scarce. Semaglutide has been shown to improve liver status in mice via the mTOR pathway [84]. Furthermore, limited evidence is available on the influence of the glucagon receptor on renal and gastrointestinal metabolism and the urea cycle, as well as on the interaction of lipid-induced hepatic intolerance. Finally, in animal studies, mainly healthy animals were used, which makes translational assumptions about diseases, e.g., MASLD and T2D, difficult. Diet, physical activity, body composition and microbiome are key parametes in metabolic dysfunction. The epigenetic aspect of diet and physical activity and the interplay with the microbiome are critical players in the outcome of metabolic dysfunction including alpha cell function and glucagon regulation yet up to date no research has been completed in this interaction.

## 5. Conclusions and Further Perspectives

With this evidence map, we aimed to identify the essential evidence gaps throughout the current literature on LACA. Longitudinal studies are needed to determine whether there are differences in the pathophysiology of LACA between adults and young patients with MASLD and related metabolic diseases. Future studies should further explore the role of the LACA across the continuum from normoglycemia to T2D across the lifespan from childhood onward. Promising pharmacological treatments have recently been identified (e.g., the GLP-1 analogs liraglutide and semaglutide and the GLP-1/GIP coagonist tirzepatide). Untangling the metabolic pathways regulating LACA with the help of omics and gene expression analysis may provide new insights into metabolic diseases and allow the identification of risk factors to potentially stop and reduce disease progression in individuals with obesity, MASLD syndrome and T2D at an early age.

## Figures and Tables

**Table 1:** Description of human studies in chronological order of publication and stratified by age subgroup.

First Author, Year	Study Design	Sample Size (n) (m:f, p, c)	Age	Patient Characteristics	Genetic Analysis
Adult studies					+
Aoki, 1974 [23]	Case–control	13 (n.a., 8, 5)	24–39	obesity	
Battezzati, 1998 [24]	Nonrandomized experimental study	7 (7:0, 0, n.a.)	24–28	lean	
Lake, 2014 [25]	Cross sectional	human liver samples 29 (n.a.)	n.a.	lean, MASLD, MASH	+
Burgos, 2016 [26]	Case–control	15 (n.a., 7, 8)	24–28	lean	
Engelbrechtsen, [27]	Randomized controlled trial	58 (n.a.)	18–65	obesity	+
Gaggini, 2018 [28]	Cross-sectional, case–control	64 (n.a., 44, 20)	35–45	lean, obesity, MASLD	
Eriksen, 2019 [29]	Case–control	46 (n.a., 20, 26)	35–40	MASLD, lean, overweight, obesity	+
Grzych, 2020 [30]	Cross sectional	112 (53:59, n.a.)	39–55	obesity, MASH, MASLD	+
Suppli, 2020 [31]	Case–control	30 (all male, n.a.)	27–53	lean, obesity, MASLD	+
Gar, 2021 [32]	Cross sectional	79 (all female, n.a.)	31–40	lean, obesity, MASLD, prediabetic	
Elovaris, 2021 [33]	Cross-sectional	15 (all males, n.a.)	20–51	lean	
Vega, 2021 [34]	Cross-sectional	33 (10:23, 23:10)	39–52	overweight, obesity	+
Svane, 2022 [35]	Randomized, placebo controlled	14 (all males, n.a.)	28–50	overweight, obesity, MASLD	
Pediatric studies					
Goffredo, 2017 [36]	Case–control	78 (38:40, 30, 48)	9–16 (pediatric)	MASLD, overweight, obesity	+
Lischka, 2021 [37]	Case control	68 (52:27, 46, 33)	9–19 (pediatric)	obesity	

n = sample size, n.a. = not available, Age = range in years, m = male, f = female, p = patients, c = controls, MASLD = metabolic dysfunction-associated steatotic liver disease; MASH = metabolic dysfunction-associated Steatohepatitis.

**Table 2:** Description of the animal studies in chronological order of publication date.

First Author, Year	Sample Size (n)	Animal Type	Animal Characteristics	Genetic Analysis
Gruppuso, 1983 [38]	12	rats	lean	
Jacobs, 2001 [39]	n.a.	rats	lean	
Kimball, 2004 [40]	n.a.	rats	lean	
Watanabe, 2012 [41]	n.a.	mice	lean	+
Solloway, 2015 [42]	n.a.	mice	lean, GCGR knockout, anti-GCGR	+
Kraft, 2017 [43]	15	dogs	lean	+
Dean, 2017 [44]	n.a	mice, zebrafish	lean	+
Galsgaard, 2018 [45]	n.a.	mice	lean, homozygotes wild type, glucagon receptor knockout	+
Miller, 2018 [46]	n.a.	mice	GLS2 knockout, glucagon, glutamine	+
Galsgaard, 2019 [47]	84	mice	lean, GRA, vehicle, transgenic, wild type, DT, GCGR, obese, NAFLD	+
Galsgaard, 2020 [48]	48	mice	lean, gcg knockout	
Winther-Sorensen 2020 [49]			GRA	+
Korenfeld, 2021 [50]	n.a.	mice	n.a.	+
E1, 2021 [51]	n.a.	mice, islets	lean, obesity, GIPR knockout	
Gart, 2022 [30]	45	mice	lean, obesity	+
Honzawa, 2022 [52]	n.a.	mice, InR1G9 cells	lean, aPkCd knockout mice,	
Maruszczak, 2022 [53]	30	mice	lean	
Thymiakou, 2023 [54]	n.a.	mice	lean, HNF4a mice	+
Elmelund, 2023 [55]	n.a.	mice	Lean, GCGR mice, GCGA mice	+

n = sample size, n.a. = not available, GCGR = glucagon receptor, GLS2 = glutaminase 2, aPkCd = alpha cell specific Proteinkinase C, GCGA = long-acting glucagon analog, GCGR = glucagon receptor antibody, GCG = glucagon, GRA = glucagon receptor antagonist, DT = diphtheria toxin.

## Supplementary Materials

The following supporting information can be downloaded at: https://www.mdpi.com/article/10.3390/life14101292/s1. Table S1: Search strategy Ovid MEDLINE(R) ALL. Table S2: Search strategy Ovid Embase. Table S3: Search strategy Web of Science Core Collection.
