# Supplementary material for: Glucagon, Metabolic Dysfunction-Associated Steatotic Liver Disease and Amino Acids in Humans and Animals without Diabetes Mellitus—An Evidence Map
Source: Life (Basel). 2024 Oct 12;14(10):1292. doi: 10.3390/life14101292 (PMC11509797; doi:10.3390/life14101292)
Supplement: Supplementary file 1 [file life-14-01292-s001.zip › life-3195454-supplementary.pdf]

Supplementary Materials: Table of Content

Search strategies (Supplementary Tables S1–S3) 2
References exluded in full-text screening with reasons 7

Search strategies (Supplementary Tables S1–S3)

Supplementary Table S1: Search strategy Ovid MEDLINE(R) ALL

Search date: 2023-02-23

#	Searches
1	Non-alcoholic Fatty Liver Disease/
2	exp Diabetes Mellitus, Type 2/
3	Insulin Resistance/
4	non-alcoholic fatty liver disease.kf,tw.
5	exp obesity/
6	NAFLD.kf,tw.
7	"fatty liver, non-alcoholic".kf,tw.
8	hepatic steatosis.kf,tw.
9	((liver or hepatic) adj5 metabolism).kf,tw.
10	diabet*.kf,kw.
11	insulin resistance.kf,tw.
12	insulin sensitivity.kf,tw.
13	hyperglycem*.kf,tw.
14	(obes* or adipos*).kf,tw.
15	receptor mutation*.kf,tw.
16	or/1-15
17	Glucagon/
18	glucagon.kf,tw.
19	Receptors, Glucagon/
20	glucagon-receptor*.kf,tw.
21	(glucagon adj2 receptor*).kf,tw.
22	Glucagon-Secreting Cells/
23	alpha-cell*.kf,tw.
24	a-cell*.kf,tw.
25	endogenous glucose production.kf,tw.
26	glucose metabolism.kf,tw.
27	glucose regulation.kf,tw.
28	glucose production.kf,tw.
29	Hepatic insulin.kf,tw.
30	fasting plasma.kf,tw.
31	or/17-30
32	Amino Acids/
33	amino acid*.kf,tw.
34	Amino Acids, Branched-Chain/
35	"branched-chain amino acid*".kf,tw.

36	or/32-35
37	16 and 31 and 36

Supplementary Table S2: Search strategy Ovid Embase

Search date: 2023-02-23

#	Searches
1	nonalcoholic fatty liver/
2	non insulin dependent diabetes mellitus/
3	insulin resistance/
4	non-alcoholic fatty liver disease.kw,tw.
5	exp obesity/
6	NAFLD.kw,tw.
7	"fatty liver, non-alcoholic".kw,tw.
8	hepatic steatosis.kw,tw.
9	((liver or hepatic) adj5 metabolism).kw,tw.
10	diabet*.kw,kw.
11	insulin resistance.kw,tw.
12	insulin sensitivity.kw,tw.
13	hyperglycem*.kw,tw.
14	(obes* or adipos*).kw,tw.
15	receptor mutation*.kw,tw.
16	or/1-15
17	glucagon/
18	glucagon.kw,tw.
19	glucagon receptor/
20	glucagon-receptor*.kw,tw.
21	(glucagon adj2 receptor*).kw,tw.
22	pancreas islet alpha cell/
23	alpha-cell*.kw,tw.
24	a-cell*.kw,tw.
25	endogenous glucose production.kw,tw.
26	glucose metabolism.kw,tw.
27	glucose regulation.kw,tw.
28	glucose production.kw,tw.
29	hepatic insulin.kw,tw.
30	fasting plasma.kw,tw.
31	or/17-30
32	amino acid/
33	amino acid*.kw,tw.
34	branched chain amino acid/
35	"branched-chain amino acid*".kw,tw.
36	or/32-35
37	16 and 31 and 36

38	limit 37 to embase

Supplementary Table S3: Search strategy Web of Science Core Collection

Search date: 2023-02-23

#	Searches
1	TS=(NAFLD)
2	TS=("fatty liver, non-alcoholic")
3	TS=("hepatic steatosis")
4	TS=((liver or hepatic) NEAR/5 metabolism)
5	TS=(diabet*)
6	TS=("insulin resistance")
7	TS=("insulin sensitivity")
8	TS=(hyperglycem*)
9	TS=(obes* or adipos*)
10	TS=("receptor mutation*")
11	TS=(glucagon)
12	TS=("glucagon-receptor*")
13	TS=(glucagon NEAR/2 receptor*)
14	TS=(alpha-cell*)
15	TS=(a-cell*)
16	TS=("endogenous glucose production")
17	TS=("glucose metabolism")
18	TS=("glucose regulation")
19	TS=("glucose production")
20	TS=("hepatic insulin")
21	TS=("fasting plasma")
22	TS=("amino acid*")
23	TS=("branched-chain amino acid*")
24	#1 OR #2 OR #3 OR #4 OR #5 OR #6 OR #7 OR #8 OR #9 OR #10
25	#11 OR #12 OR #13 OR #14 OR #15 OR #16 OR #17 OR #18 OR #19 OR #20 OR #21
26	#22 OR #23
27	#24 AND #26 AND #28

References excluded in full-text screening with reasons

Reason for exclusion	Supplementary file reference
Beta-cell pathway	1-45
No liver/glucagon/amino acid	46-73
Wrong study population	74-97
Wrong comparator	98-114
Article not available	115-127
Wrong publication type	128-139
Wrong study design	140-146

1 Amiel SA, Caprio S, Sherwin RS, Plewe G, Haymond MW, Tamborlane WV. Insulin resistance of puberty: a defect restricted to peripheral glucose metabolism. *Journal of Clinical Endocrinology & Metabolism*. 1991; 72: 277-82.

2 Ang T, Bruce CR, Kowalski GM. Postprandial Aminogenic Insulin and Glucagon Secretion Can Stimulate Glucose Flux in Humans. *Diabetes*. 2019; 68: 939-46.

3 Arai K, Lee K, Berthiaume F, Tompkins RG, Yarmush ML. Intrahepatic amino acid and glucose metabolism in a D-galactosamine-induced rat liver failure model. *Hepatology*. 2001; 34: 360-71.

4 Arrieta-Cruz I, Su Y, Gutierrez-Juarez R. Suppression of Endogenous Glucose Production by Isoleucine and Valine and Impact of Diet Composition. *Nutrients*. 2016; 8: 79.

5 Azzout-Marniche D, Gaudichon C, Blouet C, Bos C, Mathe V, Huneau J*, et al.* Liver glyconeogenesis: a pathway to cope with postprandial amino acid excess in high-protein fed rats? *American Journal of Physiology-Regulatory Integrative and Comparative Physiology*. 2007; 292: R1400-R07.

6 Bassil M, Burgos S, Marliss EB, Morais JA, Chevalier S, Gougeon R. Hyperaminoacidaemia at postprandial levels does not modulate glucose metabolism in type 2 diabetes mellitus. *Diabetologia*. 2011; 54: 1810-8.

7 Baum JI, Kimball SR, Jefferson LS. Glucagon acts in a dominant manner to repress insulin-induced mammalian target of rapamycin complex 1 signaling in perfused rat liver. *American Journal of Physiology-Endocrinology and Metabolism*. 2009; 297: E410-E15.

8 Bianchi G, Marchesini G, Brunetti N, Manicardi E, Montuschi F, Chianese R*, et al.* Impaired insulin-mediated amino acid plasma disappearance in non-alcoholic fatty liver disease: a feature of insulin resistance. *Digestive & Liver Disease*. 2003; 35: 722-7.

9 Binder E, Bermudez-Silva FJ, Andre C, Elie M, Romero-Zerbo SY, Leste-Lasserre T*, et al.* Leucine Supplementation Protects from Insulin Resistance by Regulating Adiposity Levels. *PLoS ONE [Electronic Resource]*. 2013; 8: 12.

10 Boden G, Tappy L. Effects of amino acids on glucose disposal. *Diabetes*. 1990; 39: 1079-84.

11 Borba-Murad GR, Vardanega-Peicher M, Souza HM, Lopes G, Fonseca MH, Bazotte RB. Hypoglycemia induced by insulin increases hepatic capacity to produce glucose from gluconeogenic amino acids. *Acta Pharmacologica Sinica*. 1999; 20: 1083-86.

12 Borel MJ, Williams PE, Jabbour K, Levenhagen D, Kaizer E, Flakoll PJ. Parenteral glutamine infusion alters insulin-mediated glucose metabolism. *Jpen: Journal of Parenteral & Enteral Nutrition*. 1998; 22: 280-5.

13 Brockman RP, Bergman EN, Joo PK, Manns JG. Effects of glucagon and insulin on net hepatic metabolism of glucose precursors in sheep. *American Journal of Physiology*. 1975; 229: 1344-9.

14 Browning JD, Weis B, Davis J, Satapati S, Merritt M, Malloy CR*, et al.* Alterations in hepatic glucose and energy metabolism as a result of calorie and carbohydrate restriction. *Hepatology*. 2008; 48: 1487-96.
15 Burgess SC, Hausler N, Merritt M, Jeffrey FM, Storey C, Milde A*, et al.* Impaired tricarboxylic acid cycle activity in mouse livers lacking cytosolic phosphoenolpyruvate carboxykinase. *Journal of Biological Chemistry*. 2004; 279: 48941-9.
16 Burgos SA, Chevalier S, Morais JA, Lamarche M, Kellett S, Marliss EB. Acute hyperaminoacidemia does not suppress insulin-mediated glucose turnover in healthy young men. *Applied Physiology, Nutrition, & Metabolism = Physiologie Appliquee, Nutrition et Metabolisme*. 2021; 46: 397-403.
17 Caballero B, Wurtman RJ. Differential effects of insulin resistance on leucine and glucose kinetics in obesity. *Metabolism: Clinical & Experimental*. 1991; 40: 51-8.
18 Chan C, Berthiaume F, Lee K, Yarmush ML. Metabolic flux analysis of hepatocyte function in hormone- and amino acid-supplemented plasma. *Metabolic Engineering*. 2003; 5: 1-15.
19 Chevalier S, Burgess SC, Malloy CR, Gougeon R, Marliss EB, Morais JA. The greater contribution of gluconeogenesis to glucose production in obesity is related to increased whole-body protein catabolism. *Diabetes*. 2006; 55: 675-81.
20 Chevalier S, Burgos SA, Morais JA, Gougeon R, Bassil M, Lamarche M*, et al.* Protein and glucose metabolic responses to hyperinsulinemia, hyperglycemia, and hyperaminoacidemia in obese men. *Obesity*. 2015; 23: 351-8.
21 Doi M, Yamaoka I, Nakayama M, Sugahara K, Yoshizawa F. Hypoglycemic effect of isoleucine involves increased muscle glucose uptake and whole body glucose oxidation and decreased hepatic gluconeogenesis. *American Journal of Physiology-Endocrinology and Metabolism*. 2007; 292: E1683-E93.
22 Elovaris RA, Hajishafiee M, Ullrich SS, Fitzgerald PCE, Lange K, Horowitz M*, et al.* Intragastric administration of leucine and isoleucine does not reduce the glycaemic response to, or slow gastric emptying of, a carbohydrate-containing drink in type 2 diabetes. *Diabetes Research & Clinical Practice*. 2021; 171: 108618.
23 Felig P, Wahren J, Hendler R, Brundin T. Splanchnic glucose and amino acid metabolism in obesity. *Journal of Clinical Investigation*. 1974; 53: 582-90.
24 Felig P, Wahren J, Sherwin R, Palaiologos G. Amino acid and protein metabolism in diabetes mellitus. *Archives of Internal Medicine*. 1977; 137: 507-13.
25 Forlani G, Vannini P, Marchesini G, Zoli M, Ciavarella A, Pisi E. Insulin-dependent metabolism of branched-chain amino acids in obesity. *Metabolism: Clinical & Experimental*. 1984; 33: 147-50.
26 Gu XY, Al Dubayee M, Alshahrani A, Masood A, Benabdelkamel H, Zahra M*, et al.* Distinctive Metabolomics Patterns Associated With Insulin Resistance and Type 2 Diabetes Mellitus. *FRONTIERS IN MOLECULAR BIOSCIENCES*. 2020; 7.
27 Hellman B, Larsson S, Westman S. Aspects of the glucose and amino acid metabolism in the liver and the diaphragm of normal and obese-hyperglycemic mice. *Acta Physiologica Scandinavica*. 1961; 53: 330-8.
28 Hendrick GK, Frizzell RT, Williams PE, Cherrington AD. Effect of hyperglucagonemia on hepatic glycogenolysis and gluconeogenesis after a prolonged fast. *American Journal of Physiology*. 1990; 258: E841-9.
29 Higuchi N, Kato M, Miyazaki M, Tanaka M, Kohjima M, Ito T*, et al.* Potential role of branched-chain amino acids in glucose metabolism through the accelerated induction of the glucose-sensing apparatus in the liver. *Journal of Cellular Biochemistry*. 2011; 112: 30-8.

30 Joseph SK, McGivan JD. The effect of ammonium chloride and glucagon on the metabolism of glutamine in isolated liver cells from starved rats. *Biochimica et Biophysica Acta*. 1978; 543: 16-28.
31 Just PA, Charawi S, Denis RGP, Savall M, Traore M, Foretz M*, et al.* Lkb1 suppresses amino acid-driven gluconeogenesis in the liver. *Nature communications*. 2020; 11: 6127.
32 Krebs M, Brehm A, Krssak M, Anderwald C, Bernroider E, Nowotny P*, et al.* Direct and indirect effects of amino acids on hepatic glucose metabolism in humans. *Diabetologia*. 2003; 46: 917-25.
33 Kubacka J, Cembrowska P, Sypniewska G, Stefanska A. The Association between Branched-Chain Amino Acids (BCAAs) and Cardiometabolic Risk Factors in Middle-Aged Caucasian Women Stratified According to Glycemic Status. *Nutrients*. 2021; 13: 18.
34 Libert DM, Nowacki AS, Natowicz MR. Metabolomic analysis of obesity, metabolic syndrome, and type 2 diabetes: Amino acid and acylcarnitine levels change along a spectrum of metabolic wellness. *PeerJ*. 2018; 2018.
35 Moore MC, Flakoll PJ, Hsieh PS, Pagliassotti MJ, Neal DW, Monohan MT*, et al.* Hepatic glucose disposition during concomitant portal glucose and amino acid infusions in the dog. *American Journal of Physiology - Endocrinology and Metabolism*. 1998; 274: E893-E902.
36 Moore MC, Hsieh PS, Flakoll PJ, Neal DW, Cherrington AD. Differential effect of amino acid infusion route on net hepatic glucose uptake in the dog. *American Journal of Physiology - Endocrinology and Metabolism*. 1999; 276: E295-E302.
37 Pagano G, Marena S, Scaglione L, Bodoni P, Montegrosso G, Bruno A*, et al.* Insulin resistance shows selective metabolic and hormonal targets in the elderly. *European Journal of Clinical Investigation*. 1996; 26: 650-6.
38 Rossetti P, Porcellati F, Pampanelli S, Fanelli CG, Bolli GB. Effects of amino-acid stimulation on glucagon secretion in non-diabetic and type 1 diabetic subjects during hyperinsulinaemic eu-, hypo and post-hypoglycaemic hyperglycaemia. *Diabetologia*. 2006; 49: 505-06.
39 Semba RD, Gonzalez-Freire M, Moaddel R, Sun K, Fabbri E, Zhang P*, et al.* Altered Plasma Amino Acids and Lipids Associated With Abnormal Glucose Metabolism and Insulin Resistance in Older Adults. *Journal of Clinical Endocrinology & Metabolism*. 2018; 103: 3331-39.
40 Su Y, Lam TK, He W, Pocai A, Bryan J, Aguilar-Bryan L*, et al.* Hypothalamic leucine metabolism regulates liver glucose production. *Diabetes*. 2012; 61: 85-93.
41 Wada E, Kobayashi M, Kohno D, Kikuchi O, Suga T, Matsui S*, et al.* Disordered branched chain amino acid catabolism in pancreatic islets is associated with postprandial hypersecretion of glucagon in diabetic mice: Postprandial hypersecretion of glucagon in diabetes. *Journal of Nutritional Biochemistry*. 2021: 108811.
42 Wang J, Liu Y, Lian K, Shentu X, Fang J, Shao J*, et al.* BCAA Catabolic Defect Alters Glucose Metabolism in Lean Mice. *Frontiers in Physiology*. 2019; 10: 1140.
43 Wewer Albrechtsen NJ, Kuhre RE, Hornburg D, Jensen CZ, Hornum M, Dirksen C*, et al.* Circulating Glucagon 1-61 Regulates Blood Glucose by Increasing Insulin Secretion and Hepatic Glucose Production. *Cell Reports*. 2017; 21: 1452-60.
44 Xiao F, Yu J, Guo Y, Deng J, Li K, Du Y*, et al.* Effects of individual branched-chain amino acids deprivation on insulin sensitivity and glucose metabolism in mice. *Metabolism: Clinical & Experimental*. 2014; 63: 841-50.

45 Xu H, Wang Y, Kwon H, Shah A, Kalemba K, Su X*, et al.* Glucagon changes substrate preference in gluconeogenesis. *Journal of Biological Chemistry*. 2022; 298(12) (no pagination).
46 Adachi Y, De Sousa-Coelho AL, Harata I, Aoun C, Weimer S, Shi X*, et al.* l-Alanine activates hepatic AMP-activated protein kinase and modulates systemic glucose metabolism. *Molecular Metabolism*. 2018; 17: 61-70.
47 Al-Aama JY, Al Mandi HB, Salama MA, Bakur KH, Alhozali A, Mosli HH*, et al.* Detection of Secondary Metabolites as Biomarkers for the Early Diagnosis and Prevention of Type 2 Diabetes. *Diabetes Metabolic Syndrome and Obesity-Targets and Therapy*. 2019; 12: 2675-84.
48 Bassil M, Marliss EB, Morais JA, Pereira S, Chevalier S, Gougeon R. Postprandial hyperaminoacidaemia overcomes insulin resistance of protein anabolism in men with type 2 diabetes. *Diabetologia*. 2011; 54: 648-56.
49 Bloomgarden ZT, Liljenquist J, Lacy W, Rabin D. Amino acid disposition by liver and gastrointestinal tract after protein and glucose ingestion. *American Journal of Physiology*. 1981; 241: E90-9.
50 Chevalier S, Gougeon R, Kreisman SH, Cassis C, Morais JA. The hyperinsulinemic amino acid clamp increases whole-body protein synthesis in young subjects. *Metabolism-Clinical and Experimental*. 2004; 53: 388-96.
51 Gammelsaeter R, Jenstad M, Bredahl MKL, Gundersen V, Chaudhry FA. Complementary expression of SN1 and SAT2 in the islets of Langerhans suggests concerted action of glutamine transport in the regulation of insulin secretion. *Biochemical and Biophysical Research Communications*. 2009; 381: 378-82.
52 Goni L, Qi L, Cuervo M, Milagro FI, Saris WH, MacDonald IA*, et al.* Effect of the interaction between diet composition and the PPM1K genetic variant on insulin resistance and beta cell function markers during weight loss: results from the Nutrient Gene Interactions in Human Obesity: implications for dietary guidelines (NUGENOB) randomized trial. *American Journal of Clinical Nutrition*. 2017; 106: 902-08.
53 Haussinger D, Lang F, Bauers K, Gerok W. Control of hepatic nitrogen metabolism and glutathione release by cell volume regulatory mechanisms. *European Journal of Biochemistry*. 1990; 193: 891-98.
54 Honda T, Kobayashi Y, Togashi K, Hasegawa H, Iwasa M, Taguchi O*, et al.* Associations among circulating branched-chain amino acids and tyrosine with muscle volume and glucose metabolism in individuals without diabetes. *Nutrition*. 2016; 32: 531-8.
55 Jainandunsing S, Wattimena JL, Verhoeven AJ, Langendonk JG, Rietveld T, Isaacs AJ*, et al.* Discriminative Ability of Plasma Branched-Chain Amino Acid Levels for Glucose Intolerance in Families At Risk for Type 2 Diabetes. *Metabolic Syndrome & Related Disorders*. 2016; 14: 175-81.
56 Liu Z, Kim W, Chen Z, Shin YK, Carlson OD, Fiori JL*, et al.* Insulin and glucagon regulate pancreatic alpha-cell proliferation. *PLoS ONE [Electronic Resource]*. 2011; 6: e16096.
57 Mahendran Y, Jonsson A, Have CT, Allin KH, Witte DR, Jorgensen ME*, et al.* Genetic evidence of a causal effect of insulin resistance on branched-chain amino acid levels. *Diabetologia*. 2017; 60: 873-78.
58 McCommis KS, Chen Z, Fu X, McDonald WG, Colca JR, Kletzien RF*, et al.* Loss of Mitochondrial Pyruvate Carrier 2 in the Liver Leads to Defects in Gluconeogenesis and Compensation via Pyruvate-Alanine Cycling. *Cell Metabolism*. 2015; 22: 682-94.

59 McCormack SE, Shaham O, McCarthy MA, Deik AA, Wang TJ, Gerszten RE*, et al.* Circulating branched-chain amino acid concentrations are associated with obesity and future insulin resistance in children and adolescents. *Pediatric Obesity*. 2013; 8: 52-61.
60 Nishi H, Yamanaka D, Kamei H, Goda Y, Kumano M, Toyoshima Y*, et al.* Importance of Serum Amino Acid Profile for Induction of Hepatic Steatosis under Protein Malnutrition. *Scientific Reports*. 2018; 8: 5461.
61 Owei I, Umekwe N, Stentz F, Wan J, Dagogo-Jack S. Amino acid signature predictive of incident prediabetes: A case-control study nested within the longitudinal pathobiology of prediabetes in a biracial cohort. *Metabolism: Clinical & Experimental*. 2019; 98: 76-83.
62 Pereira S, Marliss EB, Morais JA, Chevalier S, Gougeon R. Insulin resistance of protein metabolism in type 2 diabetes. *Diabetes*. 2008; 57: 56-63.
63 Qiu G, Zheng Y, Wang H, Sun J, Ma H, Xiao Y*, et al.* Plasma metabolomics identified novel metabolites associated with risk of type 2 diabetes in two prospective cohorts of Chinese adults. *International Journal of Epidemiology*. 2016; 45: 1507-16.
64 Rebholz CM, Yu B, Zheng Z, Chang P, Tin A, Kottgen A*, et al.* Serum metabolomic profile of incident diabetes. *Diabetologia*. 2018; 61: 1046-54.
65 Ribel-Madsen A, Hellgren LI, Brons C, Ribel-Madsen R, Newgard CB, Vaag AA. Plasma amino acid levels are elevated in young, healthy low birth weight men exposed to short-term high-fat overfeeding. *Physiological Reports*. 2016; 4: 12.
66 Sellmann C, Jin CJ, Degen C, De Bandt J, Bergheim I. Oral Glutamine Supplementation Protects Female Mice from Nonalcoholic Steatohepatitis¹⁻³. *Journal of Nutrition*. 2015; 145: 2280-86.
67 Shulman GI, Lacy WW, Liljenquist JE, Keller U, Williams PE, Cherrington AD. Effect of glucose, independent of changes in insulin and glucagon secretion, on alanine metabolism in the conscious dog. *Journal of Clinical Investigation*. 1980; 65: 496-505.
68 Suzuki Y, Kido J, Matsumoto S, Shimizu K, Nakamura K. Associations among amino acid, lipid, and glucose metabolic profiles in childhood obesity. *BMC Pediatrics*. 2019; 19: 273.
69 Takashina C, Tsujino I, Watanabe T, Sakaue S, Ikeda D, Yamada A*, et al.* Associations among the plasma amino acid profile, obesity, and glucose metabolism in Japanese adults with normal glucose tolerance. *Nutrition & Metabolism*. 2016; 13: 5.
70 van den Berg EH, Flores-Guerrero JL, Gruppen EG, de Borst MH, Wolak-Dinsmore J, Connelly MA*, et al.* Non-Alcoholic Fatty Liver Disease and Risk of Incident Type 2 Diabetes: Role of Circulating Branched-Chain Amino Acids. *Nutrients*. 2019; 11: 26.
71 Vogelzangs N, van der Kallen CJH, van Greevenbroek MMJ, van der Kolk BW, Jocken JWE, Goossens GH*, et al.* Metabolic profiling of tissue-specific insulin resistance in human obesity: results from the Diogenes study and the Maastricht Study. *International Journal of Obesity*. 2020; 17: 17.
72 White PJ, McGarrah RW, Grimsrud PA, Tso SC, Yang WH, Haldeman JM*, et al.* The BCKDH Kinase and Phosphatase Integrate BCAA and Lipid Metabolism via Regulation of ATP-Citrate Lyase. *Cell Metabolism*. 2018; 27: 1281-+.
73 Wurtz P, Makinen VP, Soininen P, Kangas AJ, Tukiainen T, Kettunen J*, et al.* Metabolic Signatures of Insulin Resistance in 7,098 Young Adults. *Diabetes*. 2012; 61: 1372-80.
74 Almdal TP, Jensen T, Vilstrup H. Increased hepatic efficacy of urea synthesis from alanine in insulin-dependent diabetes mellitus. *European Journal of Clinical Investigation*. 1990; 20: 29-34.

75 Almdal TP, Jensen T, Vilstrup H. Control of non-insulin-dependent diabetes mellitus partially normalizes the increase in hepatic efficacy for urea synthesis. *Metabolism: Clinical & Experimental*. 1994; 43: 328-32.
76 Babu AF, Csader S, Mannisto V, Tauriainen MM, Pentikainen H, Savonen K*, et al.* Effects of exercise on NAFLD using non-targeted metabolomics in adipose tissue, plasma, urine, and stool. *Scientific Reports*. 2022; 12: 6485.
77 Bozadjieva Kramer N, Lubaczeuski C, Blandino-Rosano M, Barker G, Gittes GK, Caicedo A*, et al.* Glucagon Resistance and Decreased Susceptibility to Diabetes in a Model of Chronic Hyperglucagonemia. *Diabetes*. 2021; 70: 477-91.
78 Bugajska J, Berska J, Wojcik M, Starzyk JB, Sztefko K. Metabolic Fingerprint of Turner Syndrome. *Journal of Clinical Medicine*. 2020; 9.
79 Caprio S, Tamborlane WV, Zych K, Gerow K, Sherwin RS. LOSS OF POTENTIATING EFFECT OF HYPOGLYCEMIA ON THE GLUCAGON-RESPONSE TO HYPERAMINOACIDEMIA IN IDDM. *Diabetes*. 1993; 42: 550-55.
80 Concepcion J, Chen K, Saito R, Gangoiti J, Mendez E, Nikita ME*, et al.* Identification of pathognomonic purine synthesis biomarkers by metabolomic profiling of adolescents with obesity and type 2 diabetes. *PLoS ONE [Electronic Resource]*. 2020; 15: e0234970.
81 Erion DM, Kotas ME, McGlashon J, Yonemitsu S, Hsiao JJ, Nagai Y*, et al.* cAMP-responsive element-binding protein (CREB)-regulated transcription coactivator 2 (CRTC2) promotes glucagon clearance and hepatic amino acid catabolism to regulate glucose homeostasis. *Journal of Biological Chemistry*. 2013; 288: 16167-76.
82 Galarregui C, Cantero I, Marin-Alejandre BA, Monreal JI, Elorz M, Benito-Boillos A*, et al.* Dietary intake of specific amino acids and liver status in subjects with nonalcoholic fatty liver disease: fatty liver in obesity (FLiO) study. *European Journal of Nutrition*. 2021; 60: 1769-80.
83 Grofte T, Wolthers T, Jorgensen JO, Poulsen PL, Vilstrup H, Moller N. Hepatic amino-to urea-N clearance and forearm amino-N exchange during hypoglycemic and euglycemic hyperinsulinemia in normal man. *Journal of Hepatology*. 1999; 30: 819-25.
84 House JD, Hall BN, Brosnan JT. Threonine metabolism in isolated rat hepatocytes. *American Journal of Physiology-Endocrinology and Metabolism*. 2001; 281: E1300-E07.
85 Iwasa M, Ishihara T, Mifuji-Moroka R, Fujita N, Kobayashi Y, Hasegawa H*, et al.* Elevation of branched-chain amino acid levels in diabetes and NAFL and changes with antidiabetic drug treatment. *Obesity Research & Clinical Practice*. 2015; 9: 293-7.
86 Jiang Y, Rose AJ, Sijmonsma TP, Broer A, Pfenninger A, Herzig S*, et al.* Mice lacking neutral amino acid transporter B⁰AT1 (Slc6a19) have elevated levels of FGF21 and GLP-1 and improved glycaemic control. *Molecular Metabolism*. 2015; 4: 406-17.
87 Kjeldsen SAS, Richter MM, Jensen NJ, Nilsson MSD, Heinz N, Nybing JD*, et al.* Development of a glucagon sensitivity test in humans: Pilot data and the GLUSENTIC study protocol. *Peptides*. 2023; 161 (no pagination).
88 Laurent F, Mialhe P. EFFECT OF FREE FATTY-ACIDS AND AMINO-ACIDS ON GLUCAGON AND INSULIN SECRETIONS IN NORMAL AND DIABETIC DUCKS. *Diabetologia*. 1978; 15: 313-21.
89 McLean P, Novello F. Influence of Pancreatic Hormones on Enzymes Concerned with Urea Synthesis in Rat Liver. *Biochemical Journal*. 1965; 94: 410-22.
90 Mu J, Qureshi SA, Brady EJ, Muise ES, Candelore MR, Jiang G*, et al.* Anti-diabetic efficacy and impact on amino acid metabolism of GRA1, a novel small-molecule glucagon receptor antagonist. *PLoS ONE [Electronic Resource]*. 2012; 7: e49572.

91 Muller WA, Faloona GR, Unger RH. The effect of alanine on glucagon secretion. *Journal of Clinical Investigation*. 1971; 50: 2215-8.
92 Okun JG, Rusu PM, Chan AY, Wu Y, Yap YW, Sharkie T*, et al.* Liver alanine catabolism promotes skeletal muscle atrophy and hyperglycaemia in type 2 diabetes. *Nature Metabolism*. 2021; 3: 394-409.
93 Pagliara AS, Stillings SN, Haymond MW, Hover BA, Matschinsky FM. INSULIN AND GLUCOSE AS MODULATORS OF AMINO ACID-INDUCED GLUCAGON-RELEASE IN ISOLATED PANCREAS OF ALLOXAN AND STREPTOZOTOCIN DIABETIC RATS. *Journal of Clinical Investigation*. 1975; 55: 244-55.
94 Rolland M, Skov PV, Larsen BK, Holm J, Gomez-Requeni P, Dalsgaard J. Increasing levels of dietary crystalline methionine affect plasma methionine profiles, ammonia excretion, and the expression of genes related to the hepatic intermediary metabolism in rainbow trout (Oncorhynchus mykiss). *Comparative Biochemistry and Physiology B-Biochemistry & Molecular Biology*. 2016; 198: 91-99.
95 Sunny NE, Kalavalapalli S, Bril F, Garrett TJ, Nautiyal M, Mathew JT*, et al.* Cross-talk between branched-chain amino acids and hepatic mitochondria is compromised in nonalcoholic fatty liver disease. *American Journal of Physiology - Endocrinology & Metabolism*. 2015; 309: E311-9.
96 Wewer Albrechtsen NJ, Faerch K, Jensen TM, Witte DR, Pedersen J, Mahendran Y*, et al.* Evidence of a liver-alpha cell axis in humans: hepatic insulin resistance attenuates relationship between fasting plasma glucagon and glucagonotropic amino acids. *Diabetologia*. 2018; 61: 671-80.
97 Zmazek J, Grubelnik V, Markovic R, Marhl M. Modeling the Amino Acid Effect on Glucagon Secretion from Pancreatic Alpha Cells. *Metabolites*. 2022; 12(4) (no pagination).
98 Bai X, Jia J, Kang Q, Fu Y, Zhou Y, Zhong Y*, et al.* Integrated Metabolomics and Lipidomics Analysis Reveal Remodeling of Lipid Metabolism and Amino Acid Metabolism in Glucagon Receptor-Deficient Zebrafish. *Frontiers in Cell & Developmental Biology*. 2020; 8: 605979.
99 Bishop CA, Schulze MB, Klaus S, Weitkunat K. The branched-chain amino acids valine and leucine have differential effects on hepatic lipid metabolism. *FASEB Journal*. 2020; 34: 9727-39.
100 Boden G, Wilson RM, Owen OE. Effects of chronic glucagon excess on hepatic metabolism. *Diabetes*. 1978; 27: 643-8.
101 Burrage LC, Madan S, Li X, Ali S, Mohammad M, Stroup BM*, et al.* Chronic liver disease and impaired hepatic glycogen metabolism in argininosuccinate lyase deficiency. *Jci Insight*. 2020; 5: 27.
102 Caprio S, Boulware S, Diamond M, Sherwin RS, Carpenter TO, Rubin K*, et al.* Insulin resistance: an early metabolic defect of Turner's syndrome. *Journal of Clinical Endocrinology & Metabolism*. 1991; 72: 832-6.
103 de Blaauw I, Deutz NE, Boers W, von Meyenfeldt MF. Hepatic amino acid and protein metabolism in non-anorectic, moderately cachectic tumor-bearing rats. *Journal of Hepatology*. 1997; 26: 396-408.
104 Hamberg O, Vilstrup H. Effects of glucose on hepatic conversion of aminonitrogen to urea in patients with cirrhosis: relationship to glucagon. *Hepatology*. 1994; 19: 45-54.
105 Kobayashi Y, Iwasa M, Miyachi H, Takei Y. Effect of Branched-chain Amino Acids on Iron and Glucose Metabolism in Rats with Liver Cirrhosis. *Hepatology*. 2013; 58: 951A-51A.
106 Krishna MG, Coker RH, Brooks Lacy D, Zinker BA, Halseth AE, Wasserman DH. Glucagon response to exercise is critical for accelerated hepatic glutamine metabolism and

nitrogen disposal. *American Journal of Physiology - Endocrinology and Metabolism*. 2000; 279: E638-E45.
107 Letellier G, Mok E, Alberti C, De Luca A, Gottrand F, Cuisset JM*, et al.* Effect of glutamine on glucose metabolism in children with Duchenne muscular dystrophy. *Clinical Nutrition*. 2013; 32: 386-90.
108 Liao X, Liu B, Qu H, Zhang L, Lu Y, Xu Y*, et al.* A High Level of Circulating Valine Is a Biomarker for Type 2 Diabetes and Associated with the Hypoglycemic Effect of Sitagliptin. *Mediators of Inflammation*. 2019; 2019 (no pagination).
109 McGuinness OP, Lacy DB, Eliasson K. Hyperglucagonemia and hepatic glucose metabolism during infection in the conscious dog. *American Journal of Physiology*. 1996; 270: E580-8.
110 Meinz H, Lacy DB, Ejiofor J, McGuinness OP. Alterations in hepatic gluconeogenic amino acid uptake and gluconeogenesis in the endotoxin treated conscious dog. *Shock*. 1998; 9: 296-303.
111 Menge BA, Schrader H, Ritter PR, Ellrichmann M, Uhl W, Schmidt WE*, et al.* Selective amino acid deficiency in patients with impaired glucose tolerance and type 2 diabetes. *Regulatory Peptides*. 2010; 160: 75-80.
112 Otani L, Nishi H, Koyama A, Akasaka Y, Taguchi Y, Toyoshima Y*, et al.* Low-arginine and low-protein diets induce hepatic lipid accumulation through different mechanisms in growing rats. *Nutrition & Metabolism*. 2020; 17: 60.
113 Samson M, Fehlmann M, Morin O, Dolaiskitabgi J, Freychet P. INSULIN AND GLUCAGON BINDING AND STIMULATION OF AMINO-ACID-TRANSPORT IN ISOLATED HEPATOCYTES FROM STREPTOZOTOCIN DIABETIC RATS. *Metabolism-Clinical and Experimental*. 1982; 31: 766-72.
114 Yu X, Huang Y, Hu Q, Ma L. Hyperhomocysteinemia stimulates hepatic glucose output and PEPCK expression. *Acta Biochimica et Biophysica Sinica*. 2009; 41: 1027-32.
115 Brennan MF, Aoki TT, Muller WA, Cahill GF, Jr. The role of glucagon as a catabolic hormone. *Surgical Forum*. 1974; 25: 72-4.
116 Bryce GF, Hope H, Wiggan G. AMINO-ACID INDUCED RELEASE OF INSULIN AND GLUCAGON IN GENETICALLY OBESE ZUCKER RAT. *Diabetes*. 1976; 25: 356-56.
117 Daniel PM, Pratt OE, Spargo E. AMINO-ACID PATTERNS IN THE BLOOD OF ALLOXAN DIABETIC RABBITS - RESPONSE TO GLUCAGON. *Journal of Physiology-London*. 1979; 287: P16-P17.
118 Ferraz M, Brunaldi K, Oliveira CE, Bazotte RB. Hepatic glucose production from L-alanine is absent in perfused liver of diabetic rats. *Research Communications in Molecular Pathology and Pharmacology*. 1997; 95: 147-55.
119 Gaggini M, Carli F, Rosso C, Latta VD, Ciociaro D, Marietti M*, et al.* Increased hepatic glucose production and insulin resistance in subjects with non-alcoholic fatty liver disease is associated to increased plasma concentrations of glucogenic amino acids. *Journal of Hepatology*. 2017; 66: S163-S63.
120 Gaggini M, Rosso C, Carli F, Della Latta V, Ciociaro D, Marietti M*, et al.* Increased hepatic glucose production and insulin resistance are associated to increased plasma concentrations of glucogenic amino acids in subjects with NAFLD. *Digestive and Liver Disease*. 2017; 49: E1-E1.
121 Gerok W, Schimassek H. [The effects of insulin and glucagon on amino acid metabolism in the isolation perfused liver]. *Verhandlungen der Deutschen Gesellschaft fur Innere Medizin*. 1967; 73: 267-72.

122 Inui Y, Yokote M. Effects of glucagon on amino acid metabolism in Japanese eels, Anguilla japonica. *GENERAL AND COMPARATIVE ENDOCRINOLOGY*. 1977; 33: 167-73.
123 James H, Gonsalves WI, Manjunatha S, Dasari S, Lanza IR, Klaus KA*, et al.* The Effect of Glucagon on Protein Catabolism During Insulin Deficiency: Exchange of Amino Acids Across Skeletal Muscle and the Splanchnic Bed. *Diabetes*. 2022; 71: 1636-48.
124 Krebs M, Brehm A, Krssak M, Anderwald C, Nowotny P, Chandramouli V*, et al.* Effects of amino acids on hepatic glucose metabolism. *Diabetes*. 2002; 51: A346-A47.
125 Miller LL. DIRECT EFFECTS OF GLUCAGON ON PROTEIN AND AMINO-ACID METABOLISM IN ISOLATED PERFUSED RAT-LIVER - INTERACTIONS WITH INSULIN AND DEXAMETHASONE IN NET SYNTHESIS OF ALBUMIN AND ACUTE-PHASE PROTEINS. *Diabetes*. 1976; 25: 865-71.
126 Perez GO, Rabinovitch A, Rietberg B, Owens B, Schiff ER. Impaired glucagon-stimulated glucose output in livers of acutely uremic rats. *Journal of Laboratory & Clinical Medicine*. 1982; 99: 669-77.
127 Roden M, Krebs M, Landau BR. to: Krebs M, Brehm A, Krssak M et al. (2003) - Direct and indirect effects of amino acids on hepatic glucose metabolism in humans. Diabetologia 46 : 917-925 - Reply. *Diabetologia*. 2004; 47: 142-43.
128 Abu-Lebdeh HS, Nair KS. Protein metabolism in diabetes mellitus. *Baillieres Clinical Endocrinology & Metabolism*. 1996; 10: 589-601.
129 Andraos S, Lange K, Clifford SA, Jones B, Thorstensen EB, Wake M*, et al.* Population epidemiology and concordance for plasma amino acids and precursors in 11-12-year-old children and their parents. *Scientific Reports*. 2021; 11.
130 Burns SP, Cohen RD, Roden M, Krebs M, Landau BR. Krebs M, Brehm A, Krssak M et al. (2003) Direct and indirect effects of amino acids on hepatic glucose metabolism in humans. Diabetologia 46:917-925 [2] (multiple letters). *Diabetologia*. 2004; 47: 141.
131 Caballero B, Field SJ, Wurtman RJ. DIFFERENTIAL-EFFECTS OF INSULIN RESISTANCE ON AMINO-ACID AND GLUCOSE-METABOLISM IN OBESITY. *CLINICAL RESEARCH*. 1988; 36: A755-A55.
132 Dean ED. A Primary Role for alpha-Cells as Amino Acid Sensors. *Diabetes*. 2020; 69: 542-49.
133 Felig P, Wahren J. The liver as site of insulin and glucagon action in normal, diabetic and obese humans. *ISRAEL JOURNAL OF MEDICAL SCIENCES*. 1975; 11: 528-39.
134 Holst JJ, Wewer Albrechtsen NJ, Pedersen J, Knop FK. Glucagon and Amino Acids Are Linked in a Mutual Feedback Cycle: The Liver-alpha-Cell Axis. *Diabetes*. 2017; 66: 235-40.
135 Kabadi UM. HEPATIC REGULATION OF PANCREATIC ALPHA-CELL FUNCTION. *Metabolism-Clinical and Experimental*. 1993; 42: 535-43.
136 Kjeldsen SAS, Zraika S, Mongovin S, Hansen LH, Terzic D, Mark PD*, et al.* Neprilysin inhibition increases plasma glucagon concentrations in humans with possible implications for hepatic amino acid metabolism. *Diabetologia*. 2020; 63: S72-S72.
137 Krebs M. Amino acid-dependent modulation of glucose metabolism in humans. *European Journal of Clinical Investigation*. 2005; 35: 351-4.
138 Li H, Lee J, He C, Zou MH, Xie Z. Suppression of the mTORC1/STAT3/Notch1 pathway by activated AMPK prevents hepatic insulin resistance induced by excess amino acids (vol 306, pg E197, 2014). *AMERICAN JOURNAL OF PHYSIOLOGY-ENDOCRINOLOGY AND METABOLISM*. 2016; 311: E899-E99.
139 Wendt A, Eliasson L. Pancreatic alpha cells and glucagon secretion: Novel functions and targets in glucose homeostasis. *Current Opinion in Pharmacology*. 2022; 63 (no pagination).

140 Burns SP, Cohen RD. To: Krebs M, Brehm A, Krssak M et al. (2003) Direct and indirect effects of amino acids on hepatic glucose metabolism in humans. Diabetologia 46:917-925. *Diabetologia*. 2004; 47: 141; author reply 42-3.
141 Calbet JAL, MacLean DA. Plasma glucagon and insulin responses depend on the rate of appearance of amino acids after ingestion of different protein solutions in humans. *Journal of Nutrition*. 2002; 132: 2174-82.
142 Charlton MR, Nair KS. Role of hyperglucagonemia in catabolism associated with type 1 diabetes - Effects on leucine metabolism and the resting metabolic rate. *Diabetes*. 1998; 47: 1748-56.
143 De Bandt JP, Lim SK, Plassart F, Lucas CC, Rey C, Poupon R*, et al.* Independent and combined actions of interleukin-1 beta, tumor necrosis factor alpha, and glucagon on amino acid metabolism in the isolated perfused rat liver. *Metabolism: Clinical & Experimental*. 1994; 43: 822-9.
144 Handlogten ME, Kilberg MS. INDUCTION AND DECAY OF AMINO-ACID-TRANSPORT IN THE LIVER - TURNOVER OF TRANSPORT ACTIVITY IN ISOLATED HEPATOCYTES AFTER STIMULATION BY DIABETES OR GLUCAGON. *Journal of Biological Chemistry*. 1984; 259: 3519-25.
145 Makeeva D, Sall T, Moskvichev D, Kartsova L, Sitkin S, Vakhitov T. CE with Cu²⁺ ions and 2-hydroxypropyl-beta-cyclodextrin additives for the investigation of amino acids composition of the culture medium in a cellular model of non-alcoholic fatty liver disease. *Journal of Pharmaceutical and Biomedical Analysis*. 2022; 213 (no pagination).
146 Varoqui H, Erickson JD. Selective up-regulation of system A transporter mRNA in diabetic liver. *Biochemical and Biophysical Research Communications*. 2002; 290: 903-08.
